# Supplementary figures and images for: Identification of conserved cross-species B-cell linear epitopes in human malaria: a subtractive proteomics and immuno-informatics approach targeting merozoite stage proteins
Source: Front Immunol. 2024 Feb 9;15:1352618. doi: 10.3389/fimmu.2024.1352618 (PMC10884153; doi:10.3389/fimmu.2024.1352618)

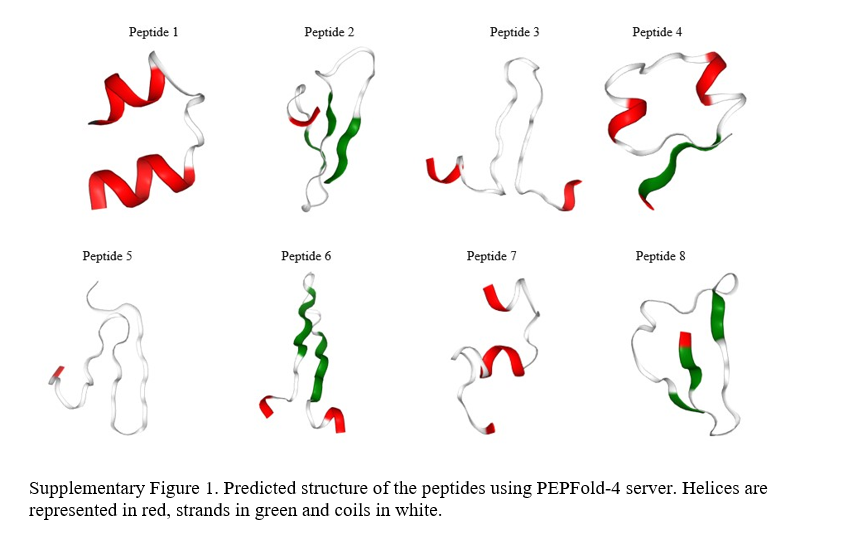

Supplement: Supplementary file 9 [file Image_1.tiff]
